# Buying Time with COVID-19 Outbreak Response, Israel

**DOI:** 10.3201/eid2609.201476

**Published:** 2020-09

**Authors:** Eyal Leshem, Arnon Afek, Yitshak Kreiss

**Affiliations:** Sheba Medical Center, Israel Ministry of Health, Tel Hashomer, Israel; Sackler School of Medicine, Tel Aviv University, Tel Aviv, Israel

**Keywords:** containment, Israel, COVID-19, travel restrictions, respiratory infections, severe acute respiratory syndrome coronavirus 2, SARS-CoV-2, SARS, 2019 coronavirus disease, zoonoses, viruses, coronavirus

## Abstract

Israel's response during the containment phase of the COVID-19 outbreak in early 2020 led to a delay in sustained community transmission and effective mitigation. During February–April 2020, a total of 15,981 confirmed cases resulted in 223 deaths. A total of 179,003 persons reported electronically to self-quarantine and were entitled to paid sick leave.

Countries’ responses to the coronavirus disease (COVID-19) emergency have been determined by their geopolitical, societal, and healthcare system characteristics. A successful response results from early identification of effective interventions tailored for these specific characteristics. At the outset of the COVID-19 outbreak response, Israel’s healthcare system faced a chronic shortage of healthcare resources; however, as Israel shifted from containment to mitigation, structural characteristics were leveraged to enhance the response. We describe Israel’s healthcare system attributes as related to geopolitical and societal status and how these factors affected the outbreak response.

Israel’s national healthcare system serves a population of 9.1 million (*1*). With 1.8 acute care hospital beds per 1,000 inhabitants and a national total of 758 licensed intensive care unit (ICU) beds, the healthcare system in Israel constantly lacks resources (*2**,**3*). In 2016, the average annual occupancy of internal medicine beds was 99% (monthly range 93%–107%) (*4*); during peak influenza season, overflow patients must receive mechanical ventilation in internal medicine wards. A shortage of surge ICU capacity during the early stages of the COVID-19 outbreak forced Israel to focus on an early aggressive containment strategy.

Israel’s life expectancy at birth of 82 years ranks eighth among Organization for Economic Cooperation and Development countries (*1**,**5*). Israel’s National Health Insurance Law (NHIL) guarantees that every legal resident receives all ambulatory and urgent medical care with very low copayment. Robust community-based healthcare services reduce the need for emergency visits to acute care medical centers. When medically indicated, all urgent care referrals and hospitalizations are free of charge. The result of these policies was that patients with suspected COVID-19 were assessed, isolated, and treated without individual hesitancy or fear of medical expenses.

During a state of national emergency, the Ministry of Health (MoH) assumes control of hospital referrals and admissions, specifically ICU hospitalizations. Stockpiles of pandemic preparedness emergency equipment, including mechanical ventilators, personal protection equipment, and critical medication, are inventoried and managed at the national level. Throughout peak COVID-19 transmission, the MoH coordinated and diverted admissions of mechanically ventilated patients to avoid overwhelming ICU capacity.

Israel’s land borders are infrequently traversed by international travels, and the country is functionally a geopolitical island. In 2019, of 4.6 million tourist entries into Israel, 88% were via international flights (*6*). Despite its culturally and politically diverse population, Israel’s society shows social cohesion, resilience, and trust in public health and government institutions.

When the COVID-19 epidemic was first reported, Israel’s MoH implemented a containment strategy that consisted of early travel restrictions to countries reporting COVID-19 transmission, as well as extensive testing and self-reported quarantine of returned travelers (Appendix Figures 1–3) (*7*). Patient contacts were identified through contact tracing and mobile phone surveillance. The MoH posted the whereabouts of confirmed cases on its website and through a mobile phone application. Persons could ascertain and report patient exposure electronically to the MoH and enter self-quarantine at home. Quarantined persons reporting electronically were issued a general statement of illness and became entitled to paid sick leave for the duration of quarantine time, enforced on employers by emergency MoH regulations and upheld by the Israel supreme court (*8*). Overall, by April 30, 2020, a total of 179,003 persons had self-reported to quarantine; 89,775 (50%) were returned travelers, and 89,228 (50%) were identified contacts of confirmed cases (*9*; Appendix Figure 4). To address increased transmission in ultra-Orthodox neighborhoods and in several Arab districts, focal lockdown measures were implemented in late April 2020. Cultural characteristics and communal responsibility led to public adherence to these sometimes onerous requirements and contributed to civil obedience.

Altogether, Israel’s containment measures proved successful in creating weeks of delay in peak transmission, more than those for some countries in Europe and cities in the United States (Figure). This delay afforded Israel’s healthcare system and the MoH time to implement preparedness measures including medical staff training, emergency department preparation for suspected patient isolation, building isolated COVID-19 units, and shifting resources to compensate for the low number of ICU beds. For example, by April 9, Sheba Medical Center in Tel Hashomer had built 327 isolated COVID-19 ICU hospitalization beds with mechanical ventilation capacity, completely separated from its 71 general (non–COVID-19) ICU beds (*10*).

**Figure:**
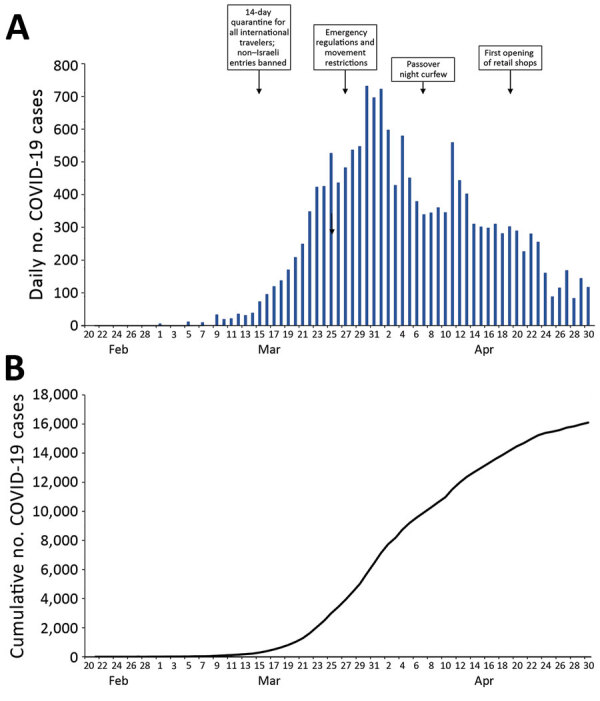
Numbers of COVID-19 cases and key public health interventions by date of implementation, Israel, February–April, 2020: A) daily numbers; B) cumulative totals.

Moving from containment to mitigation, wide-scale social distancing measures were implemented (Appendix Table). These measures included school closure, movement and travel restrictions, discontinuation of nonessential work and commerce, and complete national curfew during the holidays of Passover and Independence Day. Local curfews were instituted in neighborhoods and cities with high COVID-19 incidence and in Muslim populations during Ramadan.

Taken together, these containment and mitigation steps may have contributed to the relatively low peak incidence, low mortality rate, and preservation of healthcare system function in Israel (Figure; Appendix Table). On the basis of these experiences to date, we recommend that countries fully leverage their particular geopolitical, social, and healthcare system characteristics as soon as possible in response to crises of such magnitude.

Appendix Additional information about the response to the COVID-19 pandemic in Israel.
