# Supplementary material for: Buying Time with COVID-19 Outbreak Response, Israel
Source: Emerg Infect Dis. 2020 Sep;26(9):2251–3. doi: 10.3201/eid2609.201476 (PMC7454065; doi:10.3201/eid2609.201476)
Supplement: Appendix — Additional information about the response to the COVID-19 pandemic in Israel. [file 20-1476-Techapp-s1.pdf]

Article DOI: https://doi.org/10.3201/eid2609.201476

Buying Time with COVID-19 Outbreak Response, Israel

Appendix

Appendix Table. Public health response and key events during COVID-19 outbreak response, with number of confirmed COVID-19 cases and deaths, Israel, January–April 2020.*

Date	Event	Cumulative no. confirmed COVID-19 cases (deaths, % mortality)
2020 Jan 23	Quarantine and COVID-19 testing for symptomatic travelers from Wuhan and suspected contacts	0 (0, 0%)
2020 Jan 30	Discontinue all flights from China	0 (0, 0%)
2020 Jan 31	Land and sea borders closed to entry of non–Israeli residents	0 (0, 0%)
2020 Feb 17	All Israeli residents returning from China, Singapore, Macao, South Korea, and Thailand are required to report online and self-quarantine for 14 days	0 (0, 0%)
2020 Feb 21	All Israeli passengers from the *Diamond Princess* ship who tested negative are repatriated and quarantined at Sheba Medical Center's dedicated COVID-19 unit.<br>First COVID-19 case diagnosed in a woman quarantined after exposure on the *Diamond Princess* ship.	1 (0, 0%)
2020 Feb 26	Italy added to list of countries from which returning travelers must electronically report and self-quarantine for 14 days.<br>First COVID-19 case diagnosed in a returned traveler from Italy.	2 (0, 0%)
2020 Mar 4	France, Austria, Germany, Switzerland, and Spain added to list of countries from which self-quarantine is mandated.<br>Government and MoH workers not allowed to leave Israel.<br>Recommendation for persons >60 years of age not to leave their homes.	15 (0, 0%)
2020 Mar 8	First case of suspected community transmission with no known COVID-19 patient contacts.	41 (0, 0%)
2020 Mar 9	All persons returned from international travel required to self-quarantine for 14 days	45 (0, 0%)
2020 Mar 11	Mass events with >100 persons forbidden	97 (0, 0%)
2020 Mar 12	Borders close to all non-citizen entries.<br>Schools and universities are closed.	118 (0, 0%)
2020 Mar 14	All restaurants, shopping malls, cinemas, and sports facilities closed. Travel of >2 persons per vehicle banned.	184 (0, 0%)
2020 Mar 19	All citizen movement restricted to essential food shopping, medical needs, and work places defined as indispensable; all parks, gardens, and beaches are closed.	646 (0, 0%)
2020 Mar 20	First COVID-19 death in Israel	816 (1, 0.1%)
2020 Mar 25	Emergency regulations restrict all nonessential citizen movement >100 meters from home address; reduction of all public transportation to 25% volume; mandatory temperature measurement at workplaces; closure of synagogues	2,469 (5, 0.2%)
2020 Apr 8	Passover night; complete 4-day enhanced lockdown (effectively a curfew). Specific ban on meeting non-household persons for Passover.	9,918 (78, 0.8%)
2020 Apr 12	Mandatory facemask use at all public places	11,526 (108, 0.9%)
2020 Apr 19	First reduction in closure measures: reopening of regulated low-risk retail shops and increase to 30% of workforce	13,871 (172, 1.2%)
2020 Apr 22	Ramadan month movement restrictions implemented for 10-day period	14,688 (190, 1.3%)
2020 Apr 24	Beit Shemesh and Netivot, ultra-Orthodox cities with increased transmission, put in lockdown	15,223 (197, 1.3%)
2020 Apr 26	All street shops opened, including hairdressers and pickup from restaurants	15,471 (202, 1.3%)
2020 Apr 30	Restrictions on movement relieved.<br>Three ultra-Orthodox neighborhoods in Jerusalem and 2 Arab neighborhoods in Khura, southern Israel, put in lockdown	15,981 (223, 1.4%)

*MoH, Ministry of Health

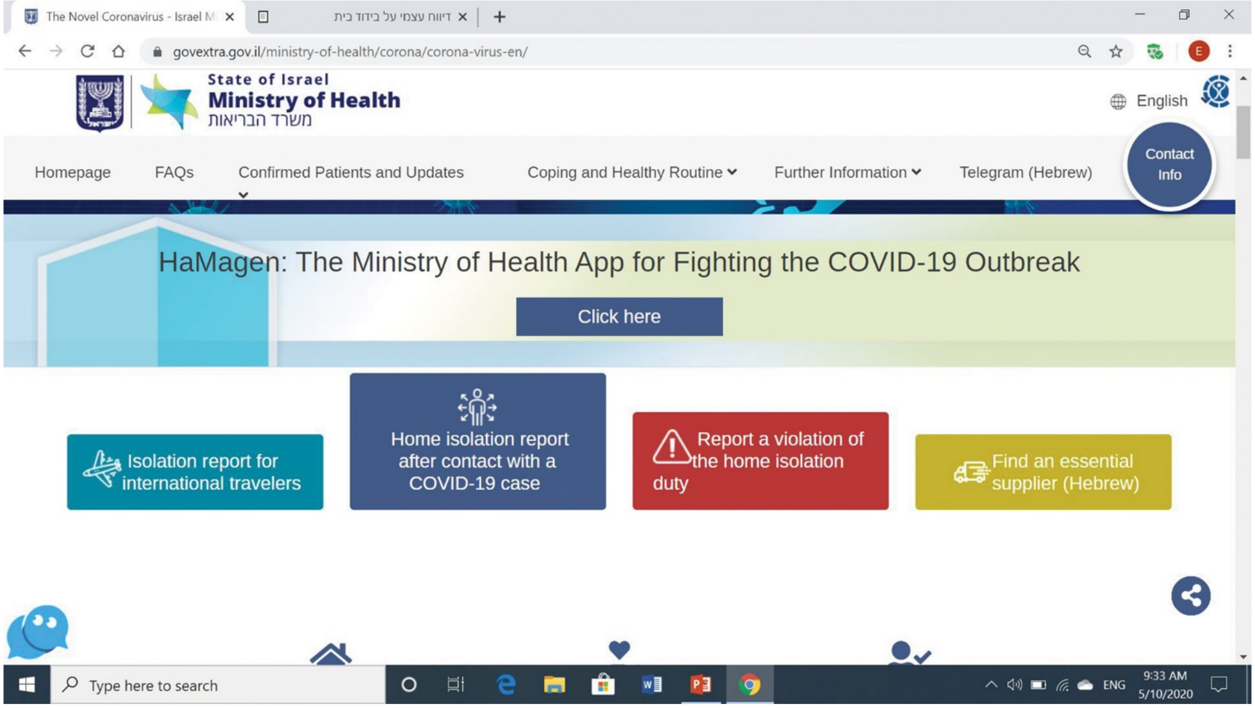

Appendix Figure 1. Webpage screenshot for self-quarantine and reporting of travel or contact with confirmed COVID-19 patient (https://govextra.gov.il/ministry-of-health/corona/corona-virus-en).

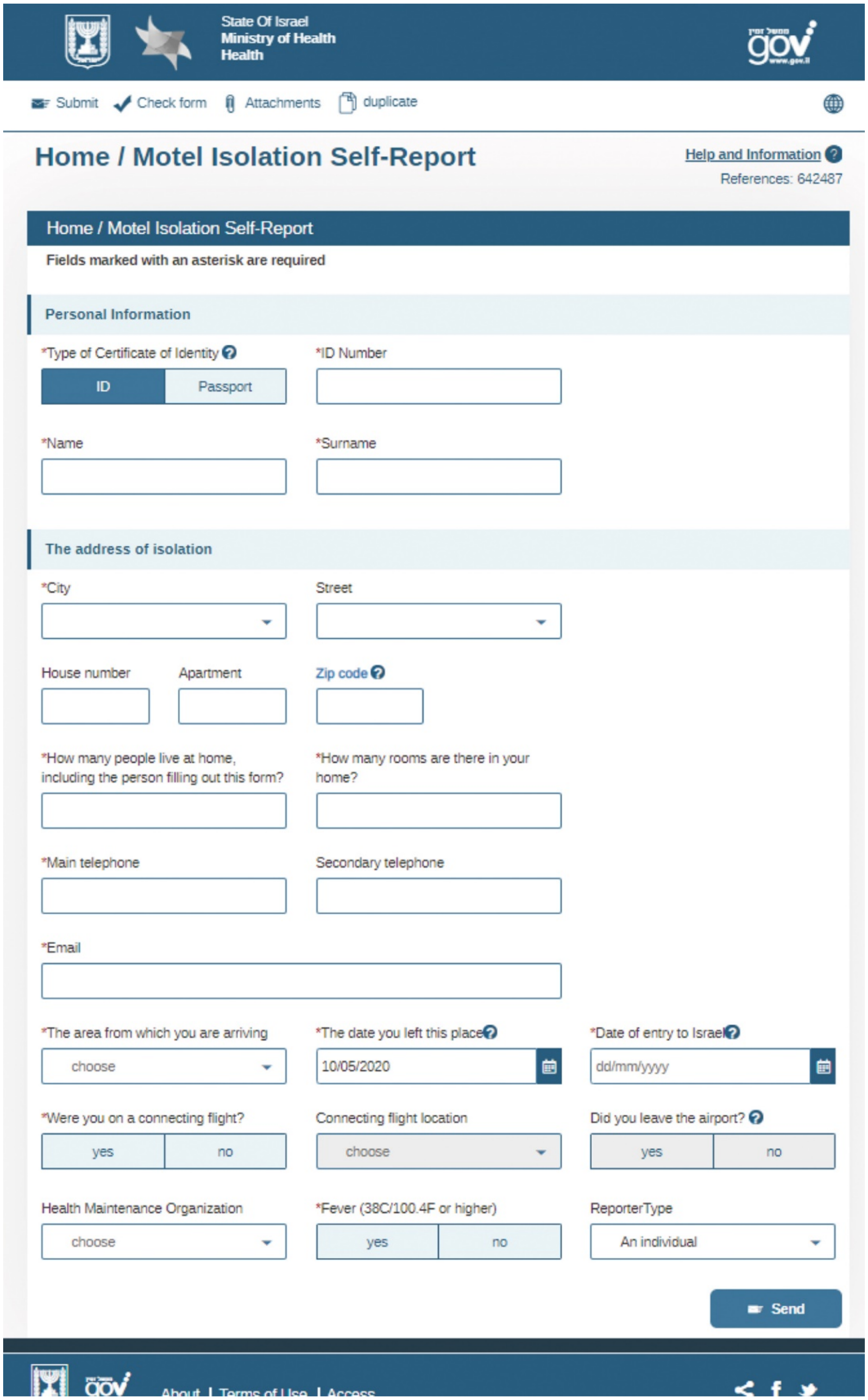

Appendix Figure 2. Electronic questionnaire webpage screenshot for self-quarantine and reporting of returned traveler in quarantine (https://govextra.gov.il/ministry-of-health/corona/corona-virus-en).

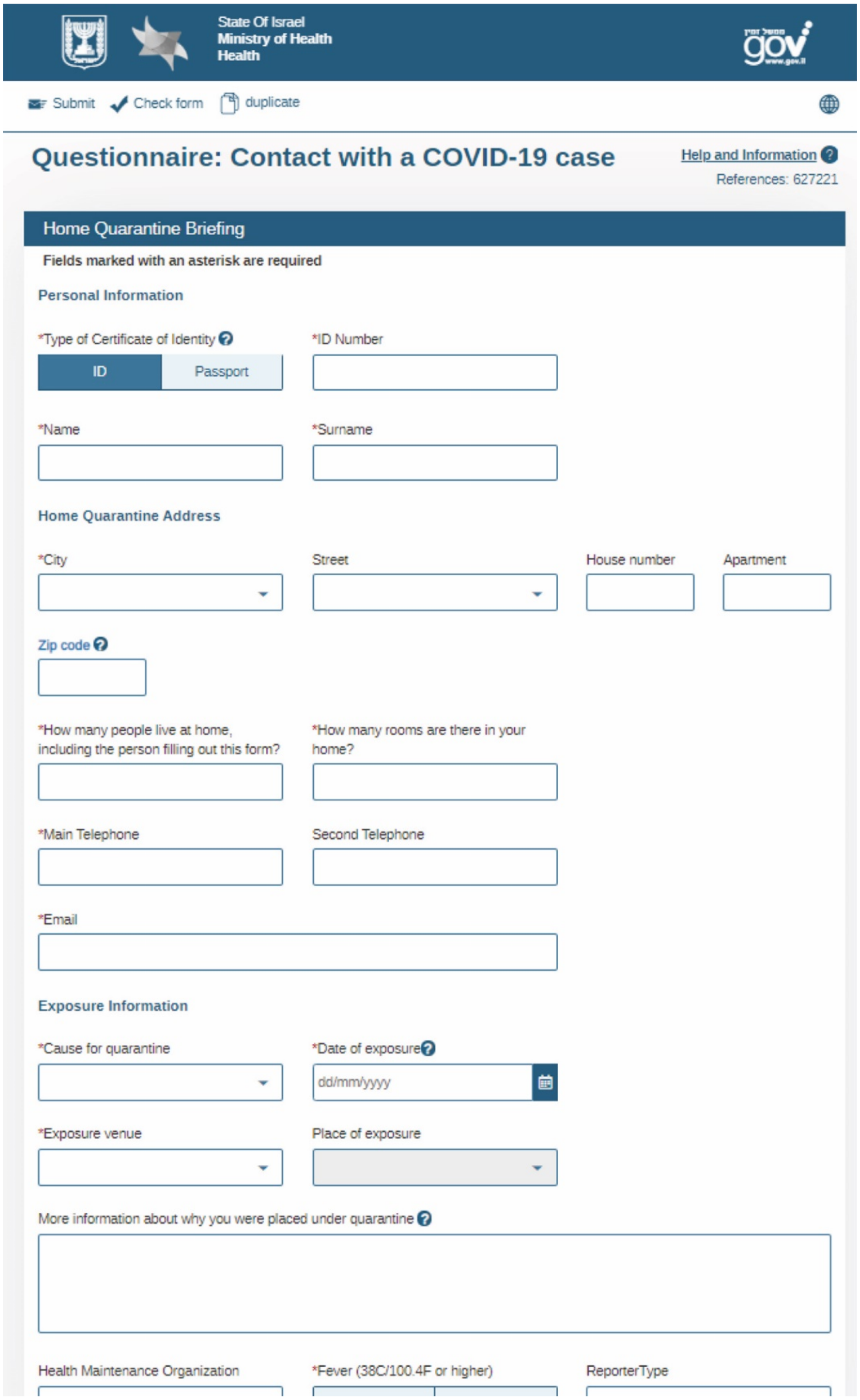
State Of Israel
Ministry of Health
Health

Submit | Check form | duplicate

Questionnaire: Contact with a COVID-19 case

Help and Information

References: 627221

Home Quarantine Briefing

Fields marked with an asterisk are required

Personal Information

*Type of Certificate of Identity: ID | Passport

*ID Number

*Name

*Surname

Home Quarantine Address

*City

Street

House number

Apartment

Zip code

*How many people live at home, including the person filling out this form?

*How many rooms are there in your home?

*Main Telephone

Second Telephone

*Email

Exposure Information

*Cause for quarantine

*Date of exposure: dd/mm/yyyy

*Exposure venue

Place of exposure

More information about why you were placed under quarantine

Health Maintenance Organization

*Fever (38C/100.4F or higher)

ReporterType

Appendix Figure 3. Electronic questionnaire webpage screenshot for self-quarantine and reporting of COVID-19 patient contact in quarantine (https://govextra.gov.il/ministry-of-health/corona/corona-virus-en).

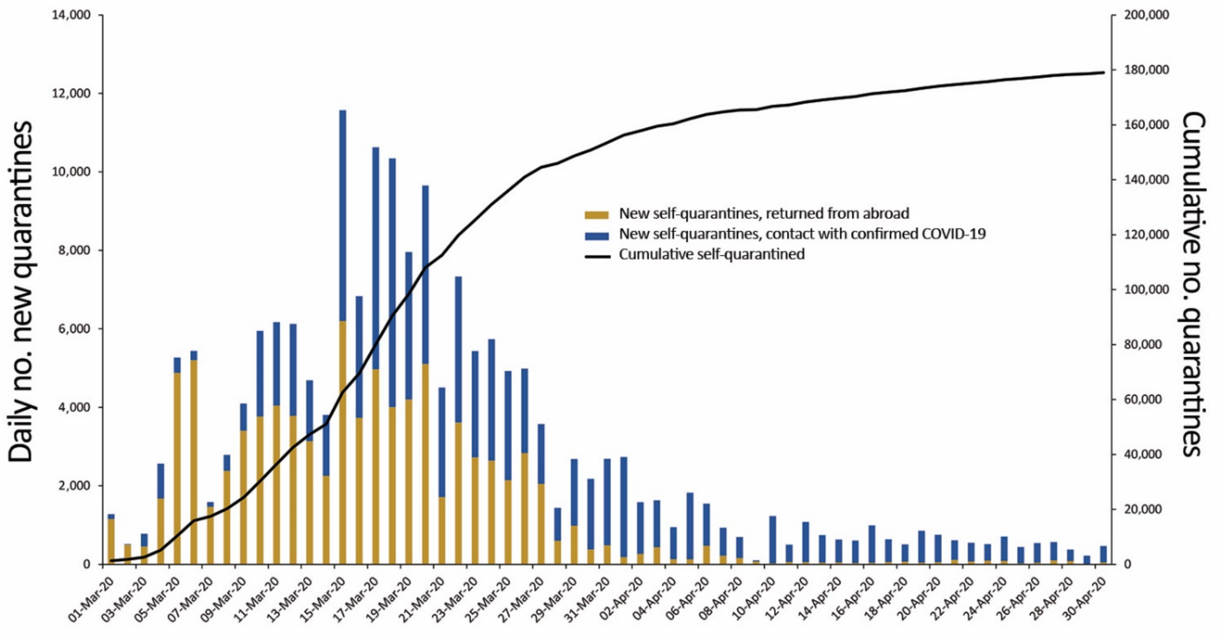

Appendix Figure 4. Daily and cumulative number of self-quarantined persons by date first self-reported and reason for quarantine (travel or case contact), Israel, March–April, 2020.
